# Overexpression of ABCB1 Transporter Confers Resistance to mTOR Inhibitor WYE-354 in Cancer Cells

**DOI:** 10.3390/ijms21041387

**Published:** 2020-02-19

**Authors:** Jingqiu Wang, Dong-Hua Yang, Yuqi Yang, Jing-Quan Wang, Chao-Yun Cai, Zi-Ning Lei, Qiu-Xu Teng, Zhuo-Xun Wu, Linguo Zhao, Zhe-Sheng Chen

**Affiliations:** 1 Department of Pharmacy, College of Chemical Engineering, Nanjing Forestry University, Nanjing 210000, China; wjq666@njfu.edu.cn; 2 Department of Pharmaceutical Sciences, College of Pharmacy and Health Sciences, St. John’s University, Queens, NY 11439, USA; yangd1@stjohns.edu (D.-H.Y.); yuqi.yang17@my.stjohns.edu (Y.Y.); jingquan.wang16@my.stjohns.edu (J.-Q.W.); chaoyun.cai16@my.stjohns.edu (C.-Y.C.); zining.lei14@my.stjohns.edu (Z.-N.L.); qiuxu.teng15@stjohns.edu (Q.-X.T.); zhuoxun.wu17@my.stjohns.edu (Z.-X.W.)

**Keywords:** ABC transporter, ABCB1, WYE-354, substrate, multidrug resistance (MDR)

## Abstract

The overexpressing ABCB1 transporter is one of the key factors leading to multidrug resistance (MDR). Thus, many ABCB1 inhibitors have been found to be able to overcome ABCB1-mediated MDR. However, some inhibitors also work as a substrate of ABCB1, which indicates that in order to achieve an effective reversal dosage, a higher concentration is needed to overcome the pumped function of ABCB1, which may concurrently increase the toxicity. WYE-354 is an effective and specific mTOR (mammalian target of rapamycin) inhibitor, which recently has been reported to reverse ABCB1-mediated MDR. In the current study, 3-(4,5-dimethylthiazolyl)-2,5-diphenyltetrazolium bromide (MTT) assay was carried out to determine the cell viability and reversal effect of WYE-354 in parental and drug-resistant cells. Drug accumulation was performed to examine the effect of WYE-354 on the cellular accumulation of chemotherapeutic drugs. The ATPase (adenosine triphosphatase) activity of the ABCB1 transporter in the presence or absence of WYE-354 was conducted in order to determine the impact of WYE-354 on ATP hydrolysis. Western blot analysis and immunofluorescence assay were used to investigate the protein molecules related to MDR. In addition, the interaction between the WYE-354 and ABCB1 transporter was investigated via in silico analysis. We demonstrated that WYE-354 is a substrate of ABCB1, that the overexpression of the ABCB1 transporter decreases the efficacy of WYE-354, and that the resistant WYE-354 can be reversed by an ABCB1 inhibitor at a pharmacological achievable concentration. Furthermore, WYE-354 increased the intracellular accumulation of paclitaxel in the ABCB1-mediated MDR cell line, without affecting the corresponding parental cell line, which indicated that WYE-354 could compete with other chemotherapeutic drugs for the ABCB1 transporter substrate binding site. In addition, WYE-354 received a high score in the docking analysis, indicating a strong interaction between WYE-354 and the ABCB1 transporter. The results of the ATPase analysis showed that WYE-354 could stimulate ABCB1 ATPase activity. Treatment with WYE-354 did not affect the protein expression or subcellular localization of the ABCB1. This study provides evidence that WYE-354 is a substrate of the ABCB1 transporter, implicating that WYE-354 should be avoided for use in ABCB1-mediated MDR cancer.

## 1. Introduction

The failure of cancer chemotherapy could result from multidrug resistance (MDR). Exploring compounds to antagonize MDR is of importance to improve the efficiency of chemotherapy [1]. ABCB1 (also known as MDR1, encoding ABCB1 gene) is a transmembrane glycoprotein that participates in the process of drug metabolism [2,3]. In a physiological state, ABCB1 can protect our bodies’ microenvironments by extruding various endogenous ligands and exogenous drugs. In a pathological condition, ABCB1 prevents drugs from crossing the blood–brain barrier into the central nervous system [4]. However, the overexpression of ABCB1 in tumor cells can result in MDR. ABCB1 is one of the subfamilies of ATP-binding cassette transporters, which is an ATP-dependent drug efflux pump with a broad substrate specificity [5]. The hydrolysis of ATP provides an energy source for ABCB1 to transport exogenous antineoplastic drugs; as a result, this reduces the intracellular drug concentration and leads to MDR [6]. Because the overexpression of ABCB1 is an important factor of MDR in tumors, inhibiting the expression and/or function of ABCB1 is the most direct way to overcome MDR in tumors. Presently, many ABCB1 inhibitors have been found, such as calcium channel blockers (verapamil) [7,8], immunosuppressants (cyclosporine A) [9,10], and antihypertensive drugs (reserpine) [11,12]. However, some of the inhibitors are the substrate of ABCB1, and the inhibition of ABCB1 requires a high serum concentration of drugs, which may be toxic, and limits the application of these inhibitors [13,14,15]. Thus, in order to overcome MDR efficiently, it is essential to identify whether the inhibitor functions as a substrate.

PI3K-AKT-mTOR signaling pathway is closely related to cell growth and proliferation [16,17]. mTOR is the central regulator of cell growth and works through two functional complexes, mTORc1 and mTORc2 [18]. In detail, mTOR promotes gene translation, ribosome formation, and autophagy to regulate cell growth, proliferation, differentiation, and survival by phosphorylating downstream 4E-BP1 and S6K1 [19,20]. As one of the important signal transduction molecules, mTOR participates in the process of cell cycle and in the occurrence and development of many diseases, and regulates many biochemical processes. Recently, mTOR has become a new target for cancer treatment [21]. Notably, it has been reported that inhibiting the phosphorylation of the PI3K/AKT/mTOR pathway could inhibit the function of the ABCB1 transporter [22,23]. WYE-354 is an effective and specific mTOR inhibitor [24,25]. It can directly combine with the kinase domain and use a catalytic effect to inhibit the effects of mTORc1 and mTORc2 [26,27]. WYE-354 inhibits protein synthesis, cell growth, and division, and has an anticancer activity by inhibiting the phosphorylation of the related downstream proteins of mTORc2, and selectively inhibiting p-AKT (S473) via an mTORc2 target in different cancer types [28]. In addition, WYE-354 can reverse ABCB1-mediated MDR [29], but whether WYE-354 is a substrate of ABCB1 is not clear. Herein, in vitro experiments were conducted to evaluate whether WYE-354 is a substrate of the ABCB1 transporter.

## 2. Results

### 2.1. The Survival Rate of ABCB1-Overexpressing Cells Treated with WYE-354 Was Higher than Those of Sensitive Cells

**Figure 1:**
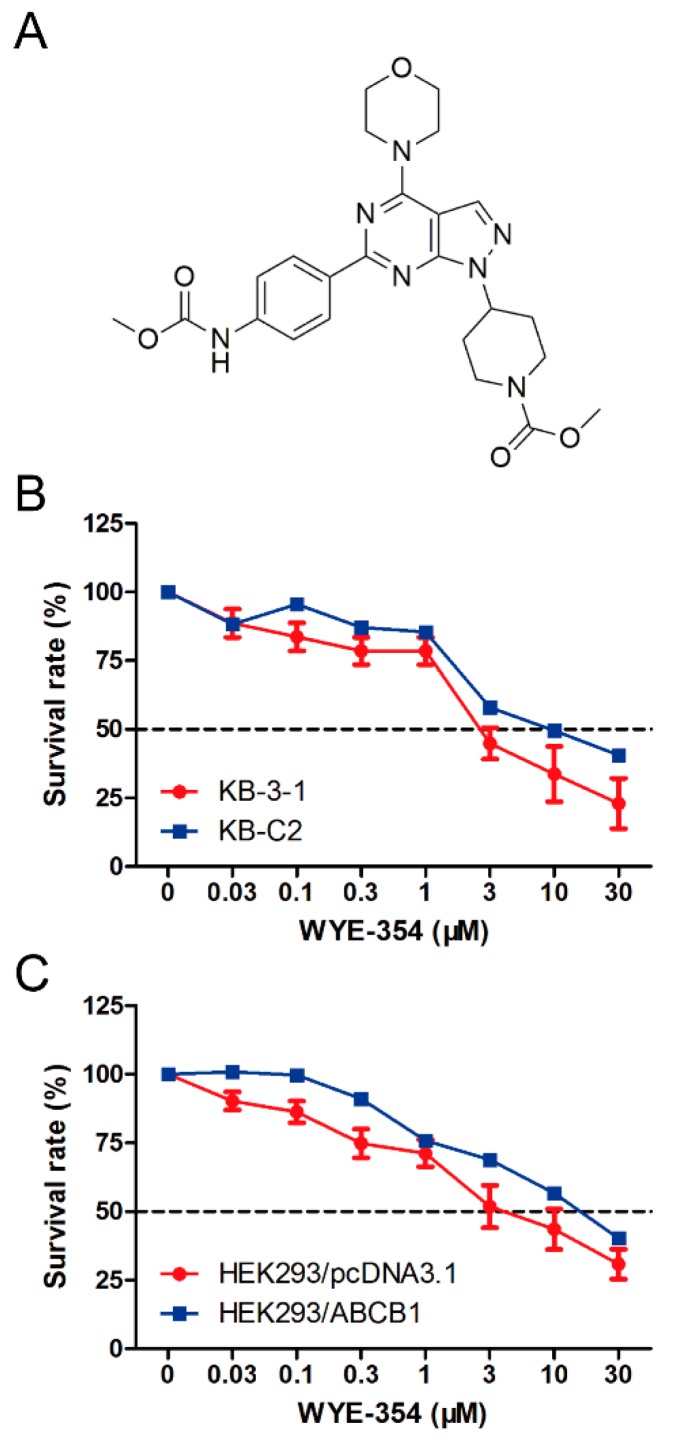
Chemical structure and survival rate of cells treated with WYE-354. (**A**) Chemical structure of WYE-354. (**B**) Cell survival–drug concentration curves for the drug-selected multidrug-resistant (MDR) cells. (**C**) ABCB1-transfented HEK293 cells. The survival rate was obtained from a formula: $\left\{\frac{{\mathrm{OD}}_{\mathrm{sample}}}{{\mathrm{OD}}_{\mathrm{control}}}\right\}\times 100\%$. The dotted line represents 50% survival.

A cell viability assay was conducted to determine the survival rate of different cell lines treated with WYE-354. As shown in Figure 1, the survival rate of KB-C2 cells treated with WYE-354 was higher compared with that in the sensitive cells (KB-3-1). Subsequently, an ABCB1-transfected HEK293 cell line was used to examine the underlying mechanism of action. It was found that the survival rate of HEK293/ABCB1 cells treated with WYE-354 was higher than that in the parental empty vector transfectant cells (HEK293/pcDNA3.1). This result indicated that ABCB1 overexpression is responsible for WYE-354-mediated drug resistance.

### 2.2. Verapamil, an ABCB1 Inhibitor, Sensitized the Efficacy of WYE-354 in ABCB1-Mediated MDR Cells

**Figure 2:**
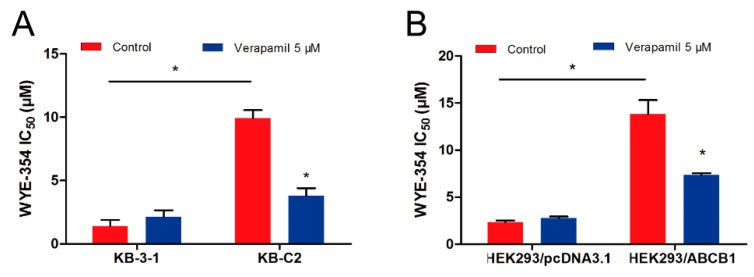
Verapamil sensitized ABCB1-overexpressing cells to WYE-354. WYE-354 co-treated with verapamil (5 μM) decreased the survival rate of WYE-354 in (**A**) KB-C2 cells and (**B**) HEK293/ABCB1 cells. All of the experiments were performed at least three times independently. Data are shown as mean ± standard deviation (SD). * represents *p* < 0.05, compared with the control group.

The pharmacological combination of WYE-354 and a known ABCB1 inhibitor verapamil was used to determine whether inhibiting the function of ABCB1 could antagonize the WYE-354-mediated drug resistance. As shown in Figure 2, WYE-354 co-treated with 5 µM verapamil decreased the survival rate of KB-C2 cells. Additionally, verapamil could restore the efficacy of WYE-354 in HEK293/ABCB1 cells. The detailed IC_50_ values are presented in Table 1. Therefore, these results indicate that WYE-354 co-treated with a reference ABCB1 inhibitor verapamil at a pharmacologically achievable concentration could decrease the survival rate of ABCB1-mediated MDR cells. The absolute OD (optical density) values of viable cells for DMSO (Dimethyl sulfoxide) and verapamil (5 µM) were 0.865 and 0.838 in KB-3-1 cells, while they are 0.933 and 0.937 in KB-C2 cells. Moreover, the absolute OD values of viable cells for DMSO and verapamil (5 µM) were 1.157 and 1.090 in HEK293/pcDNA3.1 cells, while they are 1.164 and 1.072 in HEK293/ABCB1 cells. These results suggested that verapamil at 5 µM has no significantly toxicity to both KB-3-1 and KB-C2 cells.

### 2.3. WYE-354 Stimulated ABCB1 ATPase Activity

**Figure 3:**
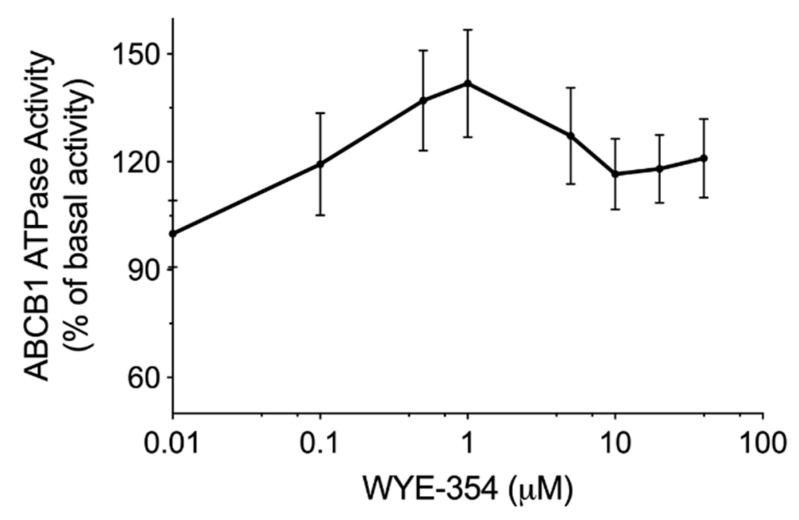
WYE-354 stimulated ABCB1 ATPase activity. The ABCB1-mediated ATP hydrolysis in the membrane vesicles was measured after incubation with WYE-354 (0–40 μM). Data are expressed as mean ± SD, obtained from three independent experiments.

An ATPase kit was used to determine the ABCB1-mediated ATP hydrolysis in the membrane vesicles after incubation with serial concentrations of WYE-354. According to the results in Figure 3, WYE-354 showed a stimulation manner on ABCB1 ATPase. The ATPase activity reached a peak of 141% of the basal activity of ABCB1.

### 2.4. WYE-354 Increased the ABCB1-Mediated Transport of [^3^H]-Paclitaxel

**Figure 4:**
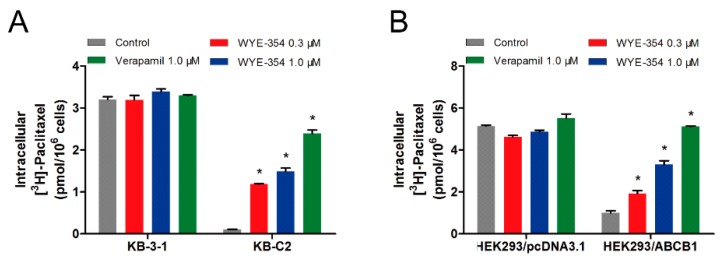
WYE-354 increased the ABCB1-mediated transport of [^3^H]-paclitaxel. (A) The effect of WYE-354 on the intracellular concentration of [^3^H]-paclitaxel in (**A**) KB-3-1 and KB-C2 cells and (**B**) HEK293/pcDNA3.1 and HEK293/ABCB1 cells after 2 h of treatment. Data are shown as mean ± SD from three independent experiments. * indicates *p* < 0.05 vs. control.

To further evaluate the mechanism of action of WYE-354, an [^3^H]-paclitaxel accumulation assay was performed to examine the drug–drug interaction between WYE-354 and paclitaxel, which is a known substrate of ABCB1. As shown in Figure 4, 1 µM of WYE-354 significantly increased the intracellular accumulation of the [^3^H]-paclitaxel in the KB-C2 cells without affecting that in the parental KB-3-1 cells. WYE-354 showed a similar effect in the ABCB1-transfected HEK293 and in its corresponding sensitive cell line. Verapamil served as a benchmark ABCB1 inhibitor. These results indicated that WYE-354 could interact competitively with other substrates at the ABCB1 transporter binding domain, which resulted in an increased accumulation of [^3^H]-paclitaxel.

### 2.5. Substrate-Drugs Co-Treated with WYE-354 Decreased the Survival Rates of ABCB1-Medified MDR Cells

**Figure 5:**
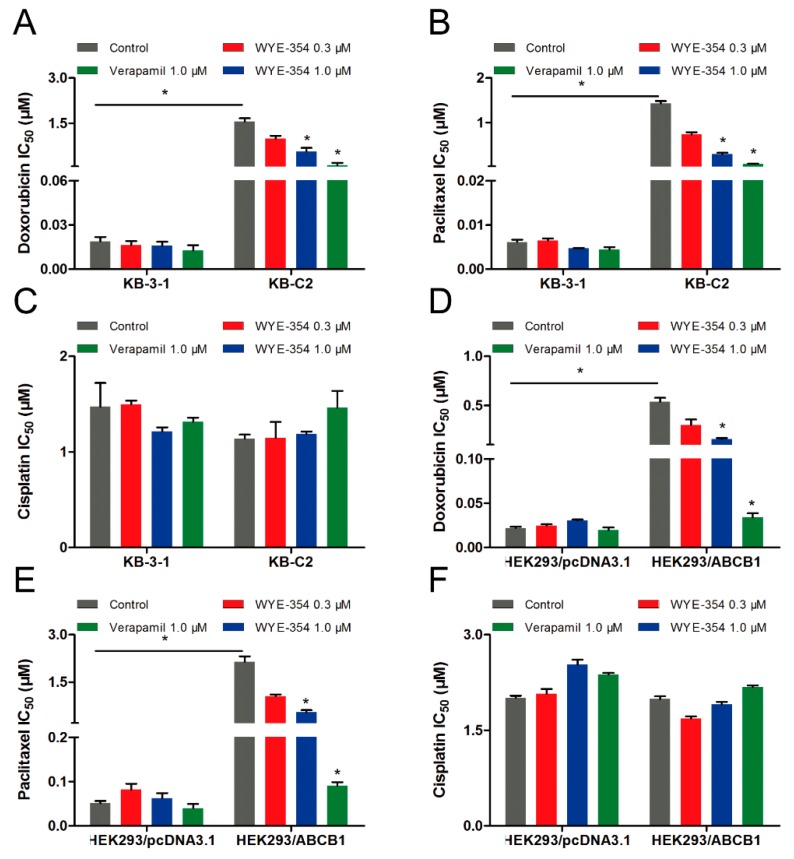
Doxorubicin and paclitaxel co-treated with WYE-354 decreased the survival rates of ABCB1-medited MDR cell lines. IC_50_ values of (**A**,**D**) doxorubicin, (**B**,**E**) paclitaxel and (**C**,**F**) cisplatin in drug-selected and ABCB1-transfected cell lines, respectively. Data were independently obtained from three experiments, shown as mean ± SD. * indicates *p* < 0.05 vs control.

Because WYE-354 could increase the [^3^H]-paclitaxel accumulation by interacting with the ABCB1 transporter competitively, we further investigated the effect of WYE-354 on the substrate-drugs of ABCB1. According to the results shown in Figure 5, doxorubicin or paclitaxel co-treated with low toxic concentrations of WYE-354 decreased the survival rates of KB-C2 cells and HEK293/ABCB1 cells, without affecting their corresponding parental cells. Moreover, WYE-354 did not significantly affect the sensitivity of all of the cell lines mentioned above to cisplatin, a non-substrate drug of ABCB1. The IC_50_ values are summarized in Table 2 and Table 3. Verapamil at 1 µM served as a benchmark inhibitor of ABCB1. These results suggested that the competitive activity of WYE-354 on the ABCB1 transporter may result in increased cytotoxicity of the ABCB1 substrate-drugs. The absolute OD values of viable cells in KB-3-1 and KB-C2 cells for DMSO, verapamil (1 µM), WYE-354 at 0.3 and 1 µM show no significant difference (for KB-3-1 cells, the absolute OD values were 1.023, 1.010, 0.864, and 0.856; for KB-C2 cells, the absolute OD values were 1.889, 1.690, 1.723, and 1.588). In addition, the absolute OD values of viable cells in HEK293/pcDNA3.1 and HEK293/ABCB1 cells for DMSO, verapamil (1 µM), and WYE-354 at 0.3 and 1 µM show no significantly difference (for HEK293/pcDNA3.1 cells, the absolute OD values were 1.927, 1.703, 1.756, and 1.716; for HEK293/ABCB1 cells, the absolute OD values were 1.426, 1.399, 1.216, and 1.250). These results suggested that verapamil and WYE-354 at 1 µM have no significant toxicity to both drug selected KB-C2 cells and ABCB1 gene transfectant HEK293/ABCB1 cells.

### 2.6. WYE-354 Did Not Affect the Protein Expression or Subcellular Localization of ABCB1

**Figure 6:**
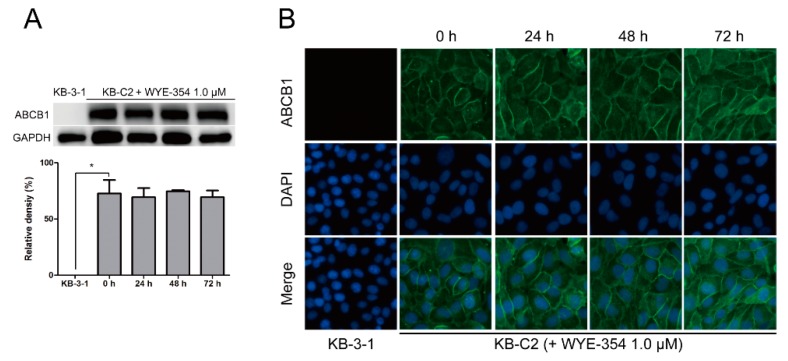
Expression level and subcellular localization of ABCB1 after up to 72 h of treatment with WYE-354. (**A**) Western blotting on the expression level of ABCB1 in the KB-C2 cells incubated with 1.0 µM WYE-354. Image J was used to quantify the relative density of each band. * *p* < 0.05, compared with the control group. (**B**) Immunofluorescence on the subcellular localization of ABCB1 in the KB-C2 cells treated with 1.0 µM WYE-354. Green—ABCB1. Blue—4′,6-diamidino-2-phenylindole (DAPI) counterstains the nuclei.

A Western blotting and immunofluorescence analysis were performed to examine the protein expression or subcellular localization of ABCB1. According to Figure 6A, after incubation with WYE-354 at 1 µM for up to 72 h, the expression level of ABCB1 (170 kDa) in KB-C2 cells was not altered. Besides, like KB-C2 cells, the expression of ABCB1 in HEK293/ABCB1 was not significantly changed after the treatment of WYE-354 for 72 h. The expression levels of ABCB1 in HEK293/ABCB1 cells are comparable to those in KB-C2 cells. This data was added as a supplemental data. In addition, as shown in Figure 6B, the localization of ABCB1 was enriched on the membranes after treated with WYE-354 at the indicated concentration for 0, 24, 48, and 72 h.

### 2.7. Molecular Docking Analysis of WYE-354 with Human ABCB1 Model

**Figure 7:**
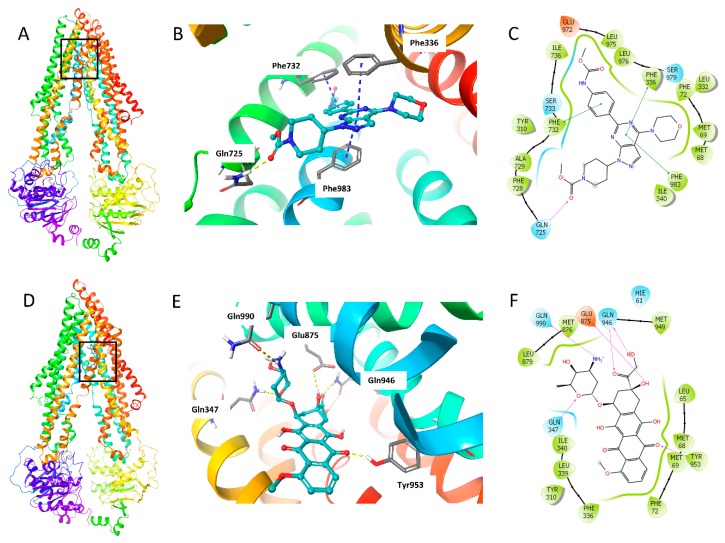
The docking simulation of WYE-354 with human ABCB1 (PDB 6QEX). (**A**) WYE-354 at the binding site of ABCB1 (indicated with a square). (**B**) WYE-354 in ball and stick mode within the binding site of human ABCB1. The atoms were colored as follows: carbon—cyan; nitrogen—blue; oxygen—red; hydrogen—white. The interactions were indicated as follows: π–π stacking—cyan dotted short line; hydrogen bonds—yellow dotted line. (**C**) The two-dimensional diagram of WYE-354 interacting with ABCB1. The amino acids within 3 Å are shown as colored bubbles, cyan indicates polar residues, and green indicates hydrophobic residues. The interactions are indicated as follows: π–π stacking—green short line; hydrogen bonds—purple arrow. (**D**) Doxorubicin at the binding site of ABCB1 (indicated with a square). (**E**) Doxorubicin in ball and stick mode within the binding site of human ABCB1. (**F**) The interaction of doxorubicin and ABCB1 in the two-dimensional diagram.

The binding mode of WYE-354 with hABCB1 mouse homology model has been reported by Ibrahim et al. [29]. In this study, we conducted the molecular docking analysis using human ABCB1 model (PDB 6QEX), which was published recently. Since we performed the assays with human cancer cells, the human ABCB1 model is more suitable for this study. The best induced-fit docking score of WYE-354 binding with a human ABCB1 model is −11.812 kcal/mol. The interactions of WYE-354 with the human ABCB1 are shown in Figure 7. Hydrogen bonding and π–π interactions are presented in the binding of WYE-354 and human ABCB1 (Figure 7B). The ester group of WYE-354 formed a hydrogen bond (-C = O⋅NH2-) with the Gln725 of the ABCB1 protein. The phenyl group of WYE-354 has a π–π stacking with a Phe732 of ABCB1, while the pyrimidine group has π–π interactions with both Phe336 and Phe983. Moreover, WYE-354 could also have hydrophobic interactions with residues of ABCB1, including Met68, Met69, Phe72, Tyr310, Leu332, Ile340, Phe728, Ala729, Leu975, and Leu976 (Figure 7C). The interaction of ABCB1 and doxorubicin, a known ABCB1 substrate, was also investigated to compare the different binding mode with WYE-354. As shown in Figure 7D, compared with WYE-354, doxorubicin interacts with the different residues of ABCB1 with a docking score of—12.241 kcal/mol. The carbonyl and hydroxyl groups in the hydroxyacetyl group form hydrogen bonds with Gln946 (Figure 7E). The amino group in the tetrahydropyran ring forms a hydrogen bond with Gln990, while the oxygen in the tetrahydropyran ring has hydrogen bonding with Gln347. One of the carbonyl groups of anthraquinone interacts with Tyr953 through a hydrogen bond. 8-hydroxyl group of 7,8,9,10-tetrahydrotetracene-5,12-dione heterocyclic ring forms hydrogen bond with Glu875. In addition, doxorubicin can also interact with some other residues of ABCB1, including Met68, Met69, Phe72, Tyr310, Phe336, Leu339, Ile340, Met876, Leu879, and Met949 (Figure 7F).

## 3. Discussion

ABCB1 is composed of 1280 amino acids expressed by a single-strand [30]. The basic structure of ABCB1 consists of four key regions, namely: two nucleoside binding domains (NBDs) and two hydrophobic transmembrane domains (TMDs) [31,32]. Each transmembrane region is composed of six transmembrane α-helices connected by hydrophilic rings, so that the ABCB1 polypeptide chains are embedded in the cell membrane. TMD as a membrane channel is conducive to drug transport, while NBD in the cytoplasm is the binding site of ATP, which is related to the energy supply for drug transport [33,34]. At present, it is documented that the major mechanism of ABCB1-mediated MDR results from ABCB1 working as a drug efflux pump. Specifically, the chemotherapeutic drugs firstly bind to ABCB1 when crossing the membrane of certain cells, and then the ATP hydrolysis provides energy, which results in pumping the drugs out of the cells; hence, this process could reduce the intracellular concentration of drugs, resulting ABCB1-mediated drug resistance [35,36,37]. Because ABCB1-mediated MDR is one of the key factors leading to the failure of chemotherapy, searching for a promising reversal method to conquer ABCB1 is still urgently needed for solving drug resistance. Nowadays, many ABCB1 inhibitors have been found [1]. Verapamil is a calcium channel blocker, which could bind to ABCB1 binding sites as a competitive substrate to be pumped out, instead of using traditional chemotherapeutic agents, as a result of reducing the drug efflux [38,39]. However, in order to achieve the reversal effect, the dosage of verapamil often exceeds the safe dose in vivo, which hinders the application of such drugs in a clinical setting [40]. WYE-354 is an effective and specific mTOR inhibitor, which has recently reported to reverse ABCB1-mediated MDR [29,41]. To our knowledge, this study is the first to provide strong evidence that WYE-354 is a substrate of ABCB1, which indicates that warning and precaution should be given, especially when WYE-354 is used as an anticancer agent.

Firstly, a cell viability assay was used to determine the cytotoxicity effect of WYE-354 in parental and ABCB1-overexpressing cell lines. All of the ABCB1-overexpressing cells showed drug resistance to WYE-354 compared with those in the corresponding parental cells. Subsequently, resistance mediated by WYE-354 could be restored with the presence of a known ABCB1 inhibitor, verapamil, which indicated that ABCB1 overexpression is a mechanism resulting from WYE-354-resistance. As the ABCB1 transporter hydrolyzes ATP and uses the energy to expel the anticancer drugs from the cells, we evaluated whether WYE-354 affected the ABCB1 ATPase activity. The results showed that the WYE-354 stimulated the activity of ABCB1 ATPase.

It is known that some inhibitors are identified as substrates that work as competitive inhibitors to reverse MDR [42]. Herein, we carried out a drug accumulation assay to investigate whether WYE-354 could compete with other substrate-chemotherapeutic drugs for the ABCB1 substrate binding site to overcome drug resistance. Additionally, in order to avoid the cytotoxicity of WYE-354, non-or low toxic concentrations of WYE-354 were chosen for this assay. According to the results, WYE-354 increased the intracellular concentration of tritium labelled antineoplastic drug in cells with ABCB1 overexpressed, without affecting sensitive cells. These results further confirmed that WYE-354 is a substrate of ABCB1, as it can compete with other substrate-drugs for the substrate binding site. Subsequently, we explored the drug-drug interaction between WYE-354 and a known substrate of ABCB1 on the substrate-drug of the ABCB1 transporter. The results show that WYE-354 increased the cytotoxic activity of doxorubicin and paclitaxel in ABCB1-mediated MDR cells, which verified that WYE-354 could compete with other substrate-drugs for the substrate binding site, and thus increased the efficacy of traditional antineoplastic agents. Furthermore, computational docking analysis was performed to help predict the interaction between WYE-354 and the ABCB1 transporter. The docking analysis simulated the molecular interactions between WYE-354 and the human ABCB1 drug-binding pocket, which then predicted the high binding affinity of WYE-354, with a docking score of—11.812 kcal/mol, which is comparable to a docking score of—12.241 kcal/mol for doxorubicin, a known substrate of ABCB1. The binding sites of human ABCB1 (PDB 6QEX) with WYE-354 are mostly π–π interactions, while doxorubicin interacts with human ABCB1 through hydrogen bonds. Moreover, the residues included in the interactions of human ABCB1 with WYE-354 and doxorubicin are different. Compared with the hABCB1 mouse homology model reported by Ibrahim et al. [29], the human ABCB1 model we used for the molecular docking analysis is more suitable for our study. Since we used human cancer cells for the assays, the human ABCB1 model can better illustrate the interaction of WYE-354 and ABCB1 within human cancer cells. Notably, it was documented that a substrate may upregulate the expression of the ABCB1 protein; hence, immunoblotting was used to determine whether WYE-354 could stimulate the expression of the ABCB1 protein under 72 h of treatment. However, WYE-354 presented no significant alteration on the expression of the ABCB1 protein. Still, long-term treatment with WYE-354 should be explored before drawing further conclusion. Moreover, the immunofluorescence results showed that WYE-354 did not change the subcellular localization of ABCB1. As drug resistance can be attribute to multiple factors, further studies are needed in order to investigate whether there are other mechanisms involved in this interaction.

## 4. Materials and Methods 

### 4.1. Chemicals

WYE-354 was purchased from MedChemExpress (MCE, Monmouth Junction, NJ, USA). Bovine serum albumin, fetal bovine serum, Dulbecco’s modified Eagle’s Medium, penicillin/streptomycin, and trypsin were purchased from Corning Incorporated (Corning, NY, USA). Verapamil was a product from Enzo Life Sciences (Farmingdale, NY, USA). [^3^H]-paclitaxel (15 Ci/mmol) was purchased from Moravek Biochemicals, Inc. (Brea, CA, USA). Doxorubicin, paclitaxel, cisplatin, the monoclonal antibodies against ABCB1 (clone F4, Cat # SAB4200775), DMSO, 3-(4,5-dimethylthiazolyl)-2,5-diphenyltetrazolium bromide (MTT), 4′,6-diamidino-2-phenylindole (DAPI), paraformaldehyde, and Triton X-100, as well as all of the other chemicals, were purchased from Sigma Chemical Co. (St. Louis, MO, USA). Horseradish peroxidase (HRP)-conjugated rabbit anti-mouse IgG secondary antibody (Cat # 7076S, lot #: 32) was obtained from Cell Signaling Technology Inc. (Danvers, MA, USA). The GAPDH loading control monoclonal antibody (GA1R) (1 mg/mL, Cat # MA5-15738, lot #: SA247966), Alexa Fluor 488 conjugated goat anti-mouse IgG cross-adsorbed secondary antibody (2 mg/mL, Cat # A32723) were obtained from Thermo Fisher Scientific Inc. (Rockford, IL, USA).

### 4.2. Cell Lines and Cell Culture

The human epidermoid carcinoma KB-3-1 and its colchicine-selected KB-C2 cell line with ABCB1 overexpressed were provided by Dr. Shin-ichi Akiyama (Kagoshima University, Kagoshima, Japan). The KB-C2 cells were maintained in a medium with 2 µg/mL colchicine [43]. HEK293/pcDNA3.1 and its ABCB1-transfected HEK293 cell line (HEK293/ABCB1) were transfected with pcDNA3.1, with empty vector or full length ABCB1. The transfected cells were selected with a medium containing 2 mg/mL G418 [44]. All of the drug-resistant cells were cultured in a medium without drugs for at least two weeks before use.

### 4.3. Cell Viability Assay

An MTT assay was used to determine the cell viability [45]. There were 5 × 10^3^ cells for each well that were seeded into 96-well plates. The next day, the cells were treated with a series of concentrations of WYE-354, or a series of concentrations of known substrates after 2 h of pretreatment with WYE-354 or the benchmark inhibitor verapamil, at indicated concentrations. After 72 h of treatment, the MTT solution (4 mg/mL) was added and incubated for another 4 h, protected from light. Finally, DMSO was added to each well after discarding the MTT solution. The OD values at 570 nm were determined with an accuSkanTM GO UV/VIS Microplate Spectrophotometer (Fisher Sci., Fair Lawn, NJ, USA).

### 4.4. [^3^H]-Paclitaxel Accumulation Assay

In brief, cells at a density of 1 × 10^5^ for each well were inoculated onto 24-well plates. The next day, WYE-354 (0.3 µM and 1 µM) or verapamil (1 µM) were added 2 h before co-incubation with [^3^H]-paclitaxel for another 2 h. After that, the cells were digested and transferred into 5 mL of a scintillation solution. A Packard TRICARB 1900CA liquid scintillation analyzer (Packard Instrument, Downers Grove, IL, USA) was used to measure the radioactivity.

### 4.5. ABCB1 ATPase Assay

As previously described [46], an ATPase assay kit from BD Biosciences (San Jose, CA, USA) was used to measure the ATPase activity of ABCB1. Briefly, 20 μg membranes were incubated in an assay buffer. Then, WYE-354 (0–40 µM) was incubated with membranes for 3 min. The ATP hydrolysis was initialized by 5 mM of Mg-ATP, while being terminated by 5% SDS (Sodium dodecyl sulfate). Subsequently, a spectrophotometer was used to measure absorption at 880 nm.

### 4.6. Western Blotting Analysis

The cells were treated at the absence or presence of WYE-354 at 1 μM for 0, 24, 48, and 72 h. The protein samples were separated by SDS-polyacrylamide gel electrophoresis, and then transferred onto the PVDF (Polyvinylidene fluoride) membrane. After 2 h of blocking with non-fat milk, the membrane was incubated with an antibody against ABCB1 or GAPDH (dilution 1:1000) at 4 °C. The next day, the membranes were incubated with an HRP-labeled antibody (dilution 1:1000) for 2 h at room temperature, and detected by electrochemiluminescence. Photographs were taken and the relative band density was analyzed by Image J.

### 4.7. Immunofluorescence Assay

Cells (2 × 10^4^ cells per well) were seeded into 24-well plates. Then, 1 µM WYE-354 was used to treat for 0, 24, 48, and 72 h. After treatment with WYE-354, the cells were fixed in 4% polyformaldehyde and permeated with 0.25% Triton X-100. Then, 6% BSA (bovine serum albumin) was used to block the cells. After being incubated with an antibody against ABCB1 (dilution 1:1000) overnight at 4 °C, the cells were incubated with an IgG antibody (dilution 1:1000) in darkness for 2 h. The nuclei of the cells were stained using DAPI (1 µg/mL). A fluorescence microscope (Life Technologies Co., Gaithersburg, MD, USA) was used to collect immunofluorescence images.

### 4.8. Molecular Modeling of Human ABCB1 Model

The molecular modeling was conducted in Maestro v11.1 (Schrödinger, LLC), as per the previous report [47]. The WYE-354 molecule and ABCB1 protein (PDB ID: 6QEX) were essentially prepared [48]. The docking grid was generated at a substrate-binding pocket of ABCB1. Glide XP docking was performed and then induced-fit docking was conducted following the default protocol.

### 4.9. Statistics

All of the studies were performed at least three times and the IC_50_ values are mean ± standard deviation (SD) calculated from at least three independent experiments. All of the data are represented as mean ± SD and analyzed by one-way analysis of variance (ANOVA), followed by the Dunnett’s test. Curve plotting and statistical analysis were performed with GraphPad Prism 8.00 (La Jolla, CA, USA) software. *p* < 0.05 was considered a significant difference compared with the corresponding control group.

## 5. Conclusions

To our knowledge, this study is the first provide evidence that WYE-354 is a substrate of the ABCB1 transporter. Moreover, the ABCB1 overexpression decreased the cytotoxicity of WYE-354, and WYE-354-mediated resistance can be reversed by verapamil, a benchmark ABCB1 inhibitor. Additionally, WYE-354 could increase the intracellular concentration of the conventional antineoplastic drugs by competitively interacting with the ABCB1 transporter.

## Figures and Tables

**Table 1:** Verapamil sensitized ABCB1-overexpressing cells to WYE-354.

Cell Line	IC_50_ ± SD ^a^ (RF ^b^) (μM)	
	WYE-354	WYE-354 + Verapamil 5 µM
KB-3-1	1.410 ± 0.481 (1.00)	2.135 ± 0.522 (1.51)
KB-C2	9.937 ± 0.625 (7.05)	3.788 ± 0.604 (2.69) *
HEK293/pcDNA3.1	2.319 ± 0.208 (1.00)	2.764 ± 0.179 (1.19)
HEK293/ABCB1	13.84 ± 1.465 (5.97)	7.372 ± 0.165 (3.18) *

^a^ IC_50_ (half maximal inhibitory concentration) values represent the mean ± SD (Standard Deviation) of three independent experiments. ^b^ The resistance fold (RF) was calculated from dividing the IC_50_ values in sensitive or resistant cells by those in parental cells without verapamil. * indicates *p* < 0.05 vs. control.

**Table 2:** Substrate-drugs of ABCB1 co-treated with WYE-354 decreased the survival rates of drug-selected cells.

Treatment	IC_50_ ± SD ^a^ (RF ^b^) (μM)	
	KB-3-1	KB-C2
Doxorubicin	0.019 ± 0.004 (1.00)	1.549 ± 0.158 (81.52)
+WYE-354 (0.3 µM)	0.016 ± 0.004 (0.84)	0.983 ± 0.140 (51.74) *
+WYE-354 (1 µM)	0.016 ± 0.004 (0.84)	0.563 ± 0.164 (29.63) *
+Verapamil (1 µM)	0.013 ± 0.005 (0.68)	0.095 ± 0.012 (5.00) *
Paclitaxel	0.006 ± 0.001 (1.00)	1.432 ± 0.075 (238.67)
+WYE-354 (0.3 µM)	0.006 ± 0.002 (1.00)	0.736 ± 0.069 (122.67) *
+WYE-354 (1 µM)	0.005 ± 0.001 (0.83)	0.296 ± 0.041 (49.33) *
+Verapamil (1 µM)	0.004 ± 0.001 (0.67)	0.075 ± 0.010 (12.50) *
Cisplatin	1.472 ± 0.351 (1.00)	1.139 ± 0.060 (0.77)
+WYE-354 (0.3 µM)	1.498 ± 0.054 (1.02)	1.148 ± 0.232 (0.78)
+WYE-354 (1 µM)	1.214 ± 0.061 (0.82)	1.187 ± 0.037 (0.81)
+Verapamil (1 µM)	1.314 ± 0.063 (0.89)	1.462 ± 0.306 (1.02)

^a^ IC_50_ values are represented as mean ± SD from three independent experiments in triplicate. ^b^ The resistance fold (RF) was calculated by dividing the IC_50_ values in KB-3-1 or KB-C2 cells by those in KB-3-1 cells without WYE-354 or verapamil. * indicates *p* < 0.05.

**Table 3:** Substrate-drugs of ABCB1 co-treated with WYE-354 decreased the survival rates of ABCB1-transfected cells.

Treatment	IC_50_ ± SD ^a^ (RF ^b^) (μM)	
	HEK293/pcDNA3.1	HEK293/ABCB1
Doxorubicin	0.022 ± 0.002 (1.00)	0.539 ± 0.054 (24.50)
+WYE-354 (0.3 µM)	0.023 ± 0.002 (1.04)	0.302 ± 0.077 (13.73) *
+WYE-354 (1 µM)	0.030 ± 0.001 (1.36)	0.157 ± 0.014 (7.14) *
+Verapamil (1 µM)	0.020 ± 0.004 (0.91)	0.034 ± 0.006 (1.54) *
Paclitaxel	0.051 ± 0.007 (1.00)	2.144 ± 0.245 (42.04)
+WYE-354 (0.3 µM)	0.082 ± 0.019 (1.61)	1.048 ± 0.087 (20.55) *
+WYE-354 (1 µM)	0.063 ± 0.016 (1.23)	0.549 ± 0.087 (10.76) *
+Verapamil (1 µM)	0.040 ± 0.013 (0.78)	0.091 ± 0.011 (1.78) *
Cisplatin	2.008 ± 0.055 (1.00)	1.990 ± 0.063 (0.99)
+WYE-354 (0.3 µM)	2.075 ± 0.106 (1.03)	1.686 ± 0.046 (0.84)
+WYE-354 (1 µM)	2.531 ± 0.111 (1.26)	1.907 ± 0.056 (0.95)
+Verapamil (1 µM)	2.375 ± 0.353 (1.18)	2.179 ± 0.037 (1.08)

^a^ IC_50_ values are represented as mean ± SD from three independent experiments in triplicate. ^b^ The resistant fold (RF) was calculated by dividing the IC_50_ values of the sensitive and resistance cells by the IC_50_ of the HEK293/pcDNA3.1 cells without WYE-354 or verapamil. * indicates *p* < 0.05.

## Supplementary Materials

The supplementary materials are available online at https://www.mdpi.com/1422-0067/21/4/1387/s1.
