# Supplementary material for: Overexpression of ABCB1 Transporter Confers Resistance to mTOR Inhibitor WYE-354 in Cancer Cells
Source: Int J Mol Sci. 2020 Feb 19;21(4):1387. doi: 10.3390/ijms21041387 (PMC7073023; doi:10.3390/ijms21041387)
Supplement: Supplementary file 1 [file ijms-21-01387-s001.pdf]

Supplemental data

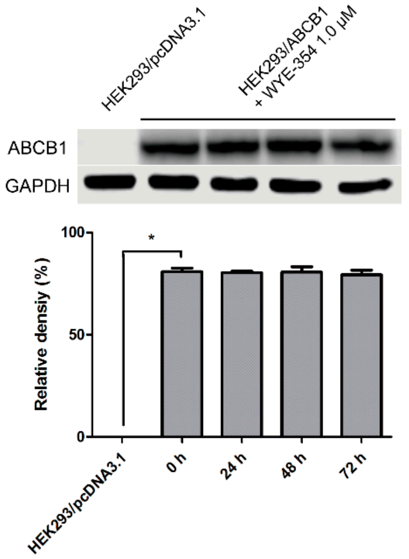

Western blotting on the expression level of ABCB1 in the HEK293/ABCB1 cells incubated with 1.0 μM WYE-354. Image J was used to quantify the relative density of each band. *$p < 0.05$, compared with the control group. Like KB-C2 cells, the expression of ABCB1 in HEK293/ABCB1 was not significantly change after the treatment of WYE-354 for 72 h
